# “Dear IOC”: Considerations for the Governance, Valuation, and Evaluation of Trends and Developments in eSports

**DOI:** 10.3389/fspor.2022.899613

**Published:** 2022-06-22

**Authors:** Dees B. W. Postma, Robby W. van Delden, Ivo M. van Hilvoorde

**Affiliations:** ^1^Human Media Interaction, University of Twente, Enschede, Netherlands; ^2^Faculty of Human Movement Sciences, Research Institute MOVE, VU University, Amsterdam, Netherlands; ^3^Department of Human Movement, School and Sport, Windesheim University of Applied Sciences, Zwolle, Netherlands

**Keywords:** esports, sports, values, ethics, governance, Olympic Games, IOC

## Abstract

In 2021, the International Olympic Committee ventured virtual space by launching their first ever *Olympic Virtual Series –* featuring virtual baseball, cycling, rowing, sailing and motor racing. Interestingly, all these virtual events take strongly after their physical counterparts. Which begs the question: Where are the massively popular esports games like Fortnite, League of Legends, and Dota?–What do the Olympic Virtual Series have that these popular video games do not? Here, we argue for the inclusion of esports within the Olympic program. In many respects, esports “act” and “behave” just like traditional sports. We argue that esports and traditional sports share many of the same values, like the values of meritocracy, competition, fair play, and the value of having a “level playing field”. Yet, in esports, many of these values remain underappreciated, losing out to negative values such as physical inactivity and game-addiction. To preserve what is worth preserving, we borrow from *Value Sensitive Design* to ameliorate the design-tensions that are foregrounded in esports. Thereby, paving possible ways toward the inclusion of esports in the Olympic program. Ultimately, the question for the IOC should not be “does it look like ‘real sport’, as we know it?”, but rather: are they sporting, rule-led, and fair activities worth preserving and setting an example for a new digitally savvy generation?

## Introduction

Only a few decades ago the worlds of sports and gaming were clearly separated. Playing Pong in the 1970s did in no way challenge our dominant ideas of sports. This has changed radically the past few decades, and electronic variants of sports (esports) are indeed challenging hegemonic–“offline”–sports. Conceptually and philosophically, there is a good case to make to consider esports a new variant of sports indeed (van Hilvoorde and Pot, 2016; Ekdahl and Ravn, 2019). Contested criteria of the definitions of sport deal with the type of organization and governance (which is primarily commercial in the case of esports) and the assumption that the skills in sports must be of a “physical” nature [e.g. (Jenny et al., 2017; Parry, 2019)].

In this paper we do not strive for a conceptual discussion on the definition of (e)sports. We do want to take this discussion a bit further. We thereby follow the assumption that esports in many ways “act” and “behave” like regular sports. Therefore, it is argued that we can and even *should* value and evaluate it as such. In esports there is training, there is coaching, there is talent identification, talent development, big money prizes to win, world championships and prestigious matched games are organized and watched worldwide. This implies as well that there are ideas of a just and fair competition, in which the winner deserves to win. And thus, there are also challenging ethical issues to deal with. In many ways, these issues mirror the perennial sport ethical issues (such as doping, match fixing), but in some other ways clearly differ from these moral issues in sport.

Notwithstanding the controversial position to analyze esports as a regular sport, we argue that the current position and worldwide popularity of esports ask for a thorough (ethical) valuation, not only of the social practice itself (the internal values of the sport and the behavior of the players), but also of its organization. The dominant view of modern esports focuses primarily on the question of the level of physical activity [e.g., (Parry, 2019, 2021)] and the risks of addiction [e.g., (Grüsser et al., 2007)]. This obscures the perspective on the intrinsic values of these games themselves, and the differentiations that can be made based on those values and meanings that these activities can have for both the players and the public. More and more, advancements in wearable technology, virtual reality, and ubiquitous computing blur the lines between the physical and the virtual world. We stipulate that (in the future) esports can be designed to fit even the most conservative definitions of sports. Thus, our aim is not to juxtapose sports with esports, but to consider the inherent values of esports and how these would fit the Olympic Movement. As such, we do not stand to question: “what is sports”, rather, we ask: “Dear IOC, what would you like your first eSport to look like?”

## Values in Olympic Sports

The new Olympic motto “higher, faster, stronger–together” (“Citius, Altius, Fortius–Communiter”) captures some of the core values in sport that many hold in high regard. Along with the three values of Olympism (excellence, respect, and friendship), the IOC stresses performance and comradery. Having a shared set of values in sports is valuable because it allows sporting organizations, athletes and other stakeholders to evaluate the changes that are inherent to the dynamical nature of sports. Sports evolve over time; new rules are instigated; new technologies arise; and new sports surface from complex socio-cultural interactions (Eichberg, 1982; Nigg, 1993). Dyer, for example, identified 11 distinct values that should be regarded when introducing novel technology to sports, inter alia: Health, naturalness, fairness, safety, and spectator appeal (Dyer, 2015, 2020). Similarly, Collins and colleagues looked at the values of sports in the context of refereeing; many values of which readily apply to esports as well, including “game fluidity” and “justice” (Collins, 2010, 2019). Most of these values identified readily apply to esports as well. According to IOC President Thomas Bach virtual sports are able to promote Olympic values indeed.

“The Olympic Virtual Series is a new, unique Olympic digital experience that aims to grow direct engagement with new audiences in the field of virtual sports. Its conception is in line with Olympic Agenda 2020+5 and the IOC's digital strategy. It encourages sports participation and promotes the Olympic values with a special focus on youth.“^1^

Following one of the recommendations of the “Olympic Agenda 2020+5 as the strategic roadmap to 2025”, it is considered “*vital that the Olympic Games continue to attract the world's best athletes in their respective sports and events and adapt to changing technologies, by considering, for example, the possibility of including physical virtual sports in future Olympic Games programmes*.” This new Olympic Virtual Series features virtual baseball, cycling, rowing, sailing and motor racing events.

Interestingly, all these esports take strongly after their physical counterparts. For the virtual cycling event, the IOC partnered up with Zwift, a platform that enables cyclists to move through virtual space by riding their home trainer. “*[I]n view of the IOC's view of sports-themed video games, along with their links to the “youth market”, video games and series such as the FIFA Series, the NBA 2K Series and Zwift may become the bases of medal events at the Olympic Games. […], the inherent characteristics of esports and the sports-themed video games make it easier to associate with the [Olympic Movement]*.” [(Abanazir, 2021), p. 11].

The question that rises when considering this line-up of virtual events is: Where are the massively popular esports games like Fortnite, League of Legends and Dota and why are these popular games excluded from the Olympic agenda? Can a line be drawn within the family of esports based on the supposed values it encourages and promotes? And if so, how can this demarcation between the “good” and the “bad” esports be legitimized? Is it indeed based upon values and sports ethics, or are other factors more relevant, such as the familiarity and resemblance with traditional sports?

Over the next couple of sections, we will consider the values of *physicality, meritocracy, competition, fair play, level playing field*, and *inclusion* and evaluate how these values are represented in traditional sports and esports. From our analysis, it will be clear that traditional sports and esports champion much of the same values in much the same way. Thus, we argue, from a values perspective, for an open attitude toward the inclusion of esports within the Olympic program. Ultimately, the question for the IOC should not be “does it look like “real sport”, as we know it?”, but rather: are they sporting, rule-led, and fair activities worth preserving and setting an example for a new digitally savvy generation?

## The Value of Physicality

Esports challenge traditional conceptions of what is sports. Consider for instance the following definition from Vossen on sports:

“Essentially, goal attainment is made impossible within all sports except by means of varying degrees of motor competency. In all games that are not sports the **physicality** of the participants is not a necessary means of prelusory goal accomplishment in that it is accessible via alternative means. In other words, **locatedness** is essential to all sports whereas it is not required of games that are not sports.” [(Vossen, 2004), p.61] -emphasis added-

Physicality and locatedness are posited as fundamental qualities of sports. Yet, both are open to interpretation. With his work on “sports over a distance”, Mueller challenged the contention that sports need to be co-located (Mueller et al., 2003a,b, 2006, 2008, 2009, 2010, 2014; Mueller and Gibbs, 2006, 2007a,b; Mueller F. et al., 2007; Mueller F. F. et al., 2007, 2009a,b). Also, commercial exercise platforms have taken flight during the corona-pandemic that show that people can engage in activities that can be rightfully called sports, from the comforts of their own home. Zwift being a prime example of that. Furthermore, physicality is not a dichotomous trait, rather it is a continuous dimension that spans across almost all human endeavors. From chess to rugby. Yet, there is a tangible and intuitive distinction to draw between chess and rugby. Whereas chess requires hardly any motor competence to excel, rugby does. Indeed, chess masters can even dispense with a physical board and pieces; playing the game entirely in their minds.

From the angle of “motor competence”, esports and rugby are more closely related to one another than are esports and Chess. *Lee Sang Hyeok, Lee Young Ho* and *Patrik Lindberg*, three of the best esports athletes at current, display intricate and precise motor movements when performing. Indeed, they lean as much on their motor skills as on their strategic and tactical insights to best their opponents. So, what level of physicality or motor complexity is required to rightfully call a game a sport? Inquisitions into the complexity of motor movement are not new. Various classification schemes have been proposed to establish the complexity of motor behavior. Probably the most influential is Gentile's taxonomy of task complexity (Gentile, 1972, 1987).

To evaluate motor skill proficiency, Gentile developed a two-dimensional taxonomy of task complexity (Table 1). At the top level, Gentile distinguished between “environment demands” and “action requirements”. Environment demands pertain to the make-up of the environment and the demands that result from it–swimming in open water imposes different demands from swimming indoors. In Gentile's taxonomy, the environmental side is further delineated by distinguishing “regulatory conditions” and “inter-trial variability”. Regulatory conditions specify whether behaviouraly relevant environmental characteristics are in motion (e.g., a teammate) or not (e.g., a goalpost). Inter-trial variability specifies whether trial-to-trial variability is present (e.g., curling) or not (e.g., 10-meter air rifle). In Gentile's taxonomy, the action requirements are delineated in a similar vein. The intended action might require the athlete to move through space (e.g., sprinting) or not (e.g., darts). And the intended action might require the athlete to manipulate an object (e.g., baseball) or not (e.g., diving). The crossing of these binary qualities leads to the classification of task complexity into 16 distinct categories, ranging from least complex in cell 1A to most complex in cell 4D (Adams, 1999; Wüest et al., 2014).

**Table 1:** Gentile's taxonomy of task complexity, including inter-trial variability and objects^a^.

			**Action Requirements**			
			**Stationary**		**In-motion**	
			***≠obj***	***=obj***	***≠obj***	***=obj***
Environment demands	Stationary	*=invar*	1A	1B	1C	1D
		*≠invar*	2A	2B	2C	2D
	In-motion	*=invar*	3A	3B	3C	3D
		*≠invar*	4A	4B	4C	4D

a *The presence or absence of inter-trial variability is denoted by ≠invar and =invar, respectively. The presence or absence of an object in the execution of a motor action is denoted as =obj and ≠obj, respectively*.

As readily illustrated above, traditional sports can be found at all levels of Gentile's taxonomy, ranging from riflery in 1B to soccer in 4D. Most esports will probably fall in the classifications 1B-4B as eSporters manipulate mouse and keyboard (or more elaborate input devices) to control the game. Depending on the nature of the game, the game might present the user with inter-trial variability (Angry Birds, 2B) or not (NBA2K-series–free throws, 1B) and / or with in-motion regulatory conditions (Counter Strike, 4B) or not (GranTourismo–Time Trial, 3B). Interestingly, in Gentile's taxonomy, input modality seems to have little effect on task complexity. Operating mouse and keyboard are considered just as complex as wielding a joystick or racing wheel. When the game-input requires users to move their body through space, the task complexity further increases [e.g., as in: (Fogtmann et al., 2011; Jensen et al., 2014, 2015; Kajastila and Hämäläinen, 2015; Sano et al., 2016; Kosmalla et al., 2017, 2018; Postma et al., 2019)]^2^. Arcadia^3^ is a prime example of an eSport that occupies the bottom-right quadrant of Gentile's taxonomy. In Arcadia, players move around in physical space, while interacting with objects in virtual reality. This mixed reality experience requires body-transport and object manipulation in an ever-changing and dynamic virtual environment. Clearly, games and esports need not be inherently less physical or complex than their traditional counterparts.

## The Value of Meritocracy and Luck

Prominent in modern-day sports is the meritocratic ideal, which puts a premium on skill, effort, and talent in the appreciation of athletic performance (Dixon, 2021). The meritocratic ideal has been the driving force behind a lot of technological innovations that allow us to value/evaluate ‘skill’ more purely. *Sports performance* is measured more accurately than ever before (e.g., photo-finish). *Sports adjudication* is more elaborate than ever before (e.g., HawkEye, VAR, Goal Line Technology). And *sports conditions* are better controlled than ever before (e.g., artificial ice rinks, indoor climbing). All to make sure that justice is being served, and that the best athlete is recognized as such [however, see: Collins (2010), Kolbinger and Lames (2017), and Collins (2019)]. Whereas scientific research actively tries to also grow beyond their laboratory settings, the sports settings quite literally are leaving the fields.

With loot-boxes, personalized power-ups, randomized spawn locations, and capricious AI-driven critters, games like Fortnite, League of Legends and Dota seem to run counter to this meritocratic ideal. Indeed, by design, such games stray away from perfectly controlled laboratory situations–allowing for serendipity and luck to influence the course of the game and the outcome thereof (Douglas and Hosoi, 2018). The game mechanics that fuel serendipity come in various flavors but phenomenologically two stand out. On the one hand, one could speak of “randomization mechanics” and on the other hand, one could speak of “balancing mechanics”. Randomization mechanics are put in place to add variance to game-play, making sure no two games are ever the same. Balancing mechanics are put in place to level the odds of different-skilled players to win. This is typically done to maximize engagement for all involved (Mueller et al., 2012; Gerling et al., 2014, 2016; Altimira et al., 2016; Jensen and Grønbæk, 2016). Below, we will argue that neither of these “mechanics” are foreign to traditional sports and that the right mix of both might actually be desirable (Kretchmar, 2012). Let us first consider *randomization mechanics*.

Randomization mechanics come in different shapes and sizes. The most basic characterization of these mechanics would be that they add “noise” or unpredictability to the natural course of the game. There can be various reasons for implementing such mechanics. Randomization adds, for example, to the replay-value of the game while discouraging inexpedient play-tactics (e.g., spawn killing or speed running). Randomization mechanics might seem foreign to traditional sports, however randomization is also clearly present in traditional sports as well (intended or unintended).

Rugby balls are egg-shaped, allowing for unpredictable ball-trajectories once the ball bounces off the ground; the natural elements, from which numerous sports draw their existence, such as surfing, (marathon) swimming and skiing cannot be controlled; and at a more abstract level some sports are simply more deterministic than others, simply by the way they are organized (Koning, 2009; Mauboussin, 2013). Using the Pythagorean theorem of statistics, Mauboussin (Mauboussin, 2013) calculated the relative contributions of luck and skill for a number of sports and placed them on a continuum. He found that sports like sprinting and tennis are more deterministic (more reliant on skill) than sports like ice hockey and football. In other words, in ice-hockey and football, there is more room for the underdog to win. In traditional sports *hope* rivals the *meritocratic* value we so much appreciate. “*[S]port verdicts, unlike outcomes in war, business, and love, do not settle things. Rather they invite both winner and loser alike to ‘play again tomorrow’*.”–[(Kretchmar, 2012), p.101]. This comes to say that there is a place both for the meritocratic values (measuring performance) and the value of hope, allowing luck to tip the scales (Dixon, 2021), however, see also: Kretchmar (1975), Dixon (1999), and Standal and Moe (2013).

Besides randomization mechanics, game developers employ *balancing mechanics* to influence game-play. Balancing mechanics in (interactive) games and sports have been studied extensively (Mueller et al., 2012; Altimira et al., 2013, 2016; Gerling et al., 2014; Jensen and Grønbæk, 2016; van Delden et al., 2017; Graf et al., 2019; Schell, 2019), and for good reason. Besides the effect of balancing mechanics on game-outcome, the proper implementation of balancing techniques is associated with increased levels of fun, engagement and self-esteem (ibid.). Balancing strategies are for example clearly present in MarioKart. Players that lag behind receive better power-ups than their adversaries. Furthermore, to keep the game close, the speed of (nearby) opponents is dynamically adjusted. This is known as rubber banding [for more detailed discussion see Schell (2019) or Tekinbas et al. (2003)]. However, more extreme measures are also part of the game-designer's arsenal. Consider the infamous “blue shell” in MarioKart–a honing missile that immobilizes the front-runner for several seconds, allowing the others to catch up. Contradicting this direction are the more popular esports games such as early releases of CS-Go where an early win resulted in serious advantages for the coming rounds rather than punishments.

Again, the deliberate implementation of balancing mechanics might be perceived to run counter to the meritocratic ideal in sports. And maybe it does, but that does not invalidate their use in esports or the claim of esports to rightfully be called sports. Balancing mechanics are also in play in traditional sports. The prime example of this stemming from cycling. In road cycling, a lesser athlete might be able to keep up with a stronger athlete by drafting behind the front-runner. Drafting is the act of reducing air-drag / wind-resistance by riding closely behind another cyclist. This effect is even more pronounced when multiple riders are driving together, such as in a peloton. Drafting allows for dramatic reductions in energy expenditure in cycling, running, speed skating and triathlons (Brisswalter and Hausswirth, 2008). Athletes make strategic use of drafting to promote (individual or team) performance (Cabaud et al., 2015; Mignot, 2015; Scelles et al., 2018). As such, drafting can be seen as a form of balancing, allowing lesser athletes to keep up with stronger athletes^4^ (Mignot, 2015). Another interesting example of balancing can be found in a recent rule change in Euroleague Basketball. Previously, when ball possession was contested by two opposing players, a tip-off situation would follow. In a tip-off situation, two players position themselves side-by-side, the ball is tossed in between them and the players jump for possession. This mechanic favors the stronger, taller, and more athletic player. Now, the alternating-possession rule is in play–granting possession to either team in an alternating fashion each time ball possession is contested anew. Finally, socio-psychological biases are known to cause referees to rule in favor of the losing team (Price et al., 2012), thereby (consciously or subconsciously) tipping the scales in favor of the weaker team.

So, what makes randomization in esports different from the inherent variability and randomness in traditional sports? And what makes balancing in esports different from balancing in traditional sports? The answer lies in the way rules, habits, practices, and interactions are created and formalized. Traditional sports tend to evolve slowly and organically over time. The nature of sports and their interactions are well-established, and novelties are implemented with care, not to harm the internal goods of a sport [cf. (Dyer, 2015, 2020)]. Games and esports on the other hand are designed and regularly updated. Nobody heard of Fortnite 10 years ago, simply because the game did not exist yet. This makes it difficult to get a feel for the nature of novel games; their balance between skill and luck; and the values that such novel games embody. Game designers will acknowledge the challenges in balancing which relates to both balancing between players and between luck and skill (Schell, 2019). This results in regular updates to fix overly advantageous strategies, for the players this relates to “a meta” the game around the game finding out the best strategy to play. We argue that these difficulties should not be weaponized to argue against esports. Considering the extant balance between *meritocracy* and *luck* in traditional sports, we argue that issues of balancing and randomization in esports are very similar, as long as luck does not obscure skill. Here, we follow Dixon: “*By definition a team does not deserve luck, which is defined as good fortune that is not due to any actions of the recipient. For this reason, I classified games whose outcome is decided by one or more instances of outrageously good or bad luck as failed athletic contests”* (Dixon, 1999). Games that minimize the potential for *failed athletic contests* are rightful candidates to deserve the sports moniker, at least from the perspective of luck and meritocratic values.

## The Value of Competition

Sports are typically understood to entail an element of competition.

“Sports so understood may be defined, or at least described, as **competitive** events involving a variety of **physical** (usually in combination with other) human skills, where the superior participant is judged to have exhibited those skills in **a superior way**.” [(Scelles et al., 2018), p.2] -emphasis added-

In a way, this quote captures the triad of considerations so far discussed: meritocracy, physicality and competition. Here, we focus on (the value of) competition. Competition is an element that distinguishes (primitive) play from sports and games. Still, as Suits would put it, “primitive play” can turn into “sophisticated play” (i.e., sports), taking either the form of judged events or refereed events (Suits, 1988). The former taking mostly the form of aesthetic sports, such as diving, figure skating and gymnastics [see also: Livingston and Forbes (2016), Livingston et al. (2020)] and the latter taking mostly the form of games, such as basketball, soccer, and rugby. As such, what is currently considered (primitive) play can turn into sports through the process of *sportification*. That is, to “*view, organize, or regulate a non-sport activity in such a way that it resembles a sport and allows a fair, pleasurable, and safe environment for individuals to compete and cooperate, and compare their performances to each other, and future and past performances*” [(Suits, 1988), p. 1].

The transformation of playful behavior into sports practice is easily illustrated (both for judged events and refereed events) by considering the immensely popular videogame *Minecraft*. Minecraft is a sandbox game without predefined, overarching goals. Players can move about freely in a procedurally generated 3D world. In the world, players can “mine” or pick up building blocks of different “materials” to build structures and crude machines. As mentioned before, the game does not present an overarching goal that needs to be attained. Rather, players are free to express themselves through the built-in game mechanics which allow for great (creative) freedom. Walking and jumping belong to the core mechanics of Minecraft. In itself, nothing special. However, the combination of these elements evolved into the practice of *Minecraft Parkour* (much akin to classic parkour), which consolidated its existence in the form of full-blown Minecraft-Parkour championships. Involving winners, losers, and extensive training. As such, basic mechanics like walking and jumping evolved from basic mechanics to “sophisticated play” through the addition of sports elements (i.e., game-rules, contestants, and prizes). Here, casual Minecraft organically turned into a refereed event. Not through the deliberate implementation of gamification elements (points or badges) but through the intrinsic reward relating to the display of skillful behavior, fueled by talent and dedication.

Another example of how the basic mechanics of Minecraft might turn into a (judged) sporting event is by considering the intricate buildings that are erected from basic building blocks^5^ Sports like diving, figure skating and gymnastics are judged based on their aesthetic value. Athletes in aesthetic sports are judged on how well they approximate an ideal (Suits, 1988). The building of intricate, artistic, and sometimes hyper-realistic, structures in Minecraft might be considered an aesthetic sporting event. One in which the contestants either express their creativity in free-form or one in which contestants approximate an ideal by replicating existing structures like the Eiffel tower. Indeed, in the early days of the Olympics, medals were awarded for architecture as well (Kates and Clapperton, 2015; Parry, 2019). Would the adjudication of the aesthetics of Minecraft buildings be so far removed from the adjudication of athletic form in gymnastics?–Yes, obviously. As the creation of beautiful buildings does not entail motor competence. Just as in chess, the builder could instruct someone else to do the actual building. This point has been addressed above. Some form of motor competence is required for a game to be considered an (e)Sport. The take-away message here is that many games *do* hold the potential to be considered a sport when striking the right balance between meritocracy, physicality, and competition.

Finally, closely related to the issue of the relative contribution of skill vs. luck, sports differ in their level of competitive balance^6^. A competition is said to be fully balanced when “*each participant starts with an equal chance of winning, so that the outcome will be completely uncertain*.” [(Szymanski, 2001), p. 1]. Both skill and luck influence competitive balance: A coin-tossing competition as well as an athletics competition might be perfectly balanced, but each for widely different reasons. In the former case, competitive balance is the result of stochastic variance, benefitting every player evenly in a way that skill has no part in the outcome. In the latter case, competitive balance is mostly the result of the relative quality of the athletes. “*Given a certain talent distribution, rules and institutions in a sport determine the translation of this variation [of talent] into variation of performance*.” [(Koning, 2009), p. 231]. It is the translation of variation that is of interest here. Games like ‘Mario Party’ are deliberately designed to maximize the variation in performance (within playable limits)–allowing players with ranging skills to enjoy competitive balance. Because sports are essentially contests of skill, such extreme translations of talent variation are undesirable. Luck-to-skill ratios and competitive balance metrics can be statistically approximated (e.g. Szymanski, 2001; Zimbalist, 2002; Koning, 2009; Livingston and Forbes, 2016; Douglas and Hosoi, 2018; Heere, 2018) and can be taken to infer the ability of a game to let skill shine through. Investigations into luck-to-skill ratios show that games such as League of Legends are actually on par with baseball and football (Douglas and Hosoi, 2018). Such comparative analyses, between esports and traditional sports, might help to decide whether the competitive balance / luck-to-skill ratio of a novel game is at the desired level for inclusion in the Olympic Games.

## The Value of Fair Play

In this paragraph we will use Zwift as a case to further explore the value of fair play and sportsmanship in esports–we further elaborate on two relevant aspects: the governance of esports in relation to cheating and the values that are transmitted or mirrored within and through the virtual sport context.

Most sports are governed by both constitutive and regulatory rules. Violation of a constitutive rule (such as the hand play of the ball by a soccer player) will result in a penalty as determined by the sport itself. Violating more regulatory rules (such as driving out of the wind behind a car when cycling) can lead to moral outrage but does not necessarily lead to a formal sanction. Distinctive for esports is that many of the regulatory rules also have been formalized and have become part of the gamification of the sport. In Zwift, in-game rewards sometimes deal with the gray area between “tactics” and “cheating”, such as getting more draft, becoming temporarily lighter in weight, or even getting a better and faster bike. As you progress through the game of Zwift, the riders gain experience points (XP) and are motivated to unlock all kinds of rewards based upon those points. The amount of training on Zwift is reflected in the upgrade of levels and is further stimulated by a variety of in-game goals, challenges, missions and badges. An example of such a badge would be the “Masochist”, that is earned when climbing the Alpe du Zwift 25 times [see also: van Delden et al. (2017)]. Challenges in the game make it possible to unlock all kinds of better equipment, such as wheels and bicycles.

At first glance, these awards seem like harmless and playful features of gamification. But things get more serious when there are official Zwift competitions organized by national sports associations. One of the first scandals involved a British cyclist who took part in the first British Cycling eRacing Championship in 2019 that was live broadcasted on British television and around the world via YouTube and Facebook. The winner had “illegally” obtained a “new” bicycle (which normally requires 50,000 meters of climbing) and was disqualified and banned from racing for 6 months. The British Cycling organization issued the following statement:

“It is stated that Cameron Jeffers participated in the British Cycling Zwift eRacing Championships qualifier on the 24 February 2019 and the British Cycling Zwift eRacing Championships Final on 28 March 2019, using in-game equipment that was obtained in a manner that contravenes the Disciplinary Rules for Cycle Sport, the Code of Conduct and the regulations stipulated in the General & eRacing Technical Regulations: This constitutes misconduct under Clauses 3.1 and 3.2 (a), (c) & (h) of the Disciplinary Rules for Cycle Sport.”^7^

It is interesting how the cyclist in question accounts for his behavior on his YouTube channel.^8^ This shows a narrative as we also know from the alleged doping user, who looks for mitigating circumstances and references to the rules that were not yet clear enough or wrongly applied. This first official application of a new rule can be understood as inherent in evolutions in a new sport in development, comparable to the introduction of the penalty kick in soccer at the end of the 19th century. Interesting and relevant for the discussion about values is the fact that this case illustrates the importance of trust: trust in the rules, the organization, the participants and the opponents. This confidence is also inherent of the appeal of the sport for a wider audience, but also for the riders themselves. One of the interviewed professionals riding on Zwift said: “*If [...] competitive ZWIFT riding is going to find its way into modern cycling after all, then something [about cheating prevention] needs to be changed*” [(Westmattelmann et al., 2021), p. 10]. Other cheating issues in Zwift involve the manipulation of body weight or manipulating the smart trainer hardware (Westmattelmann et al., 2020). Recently, just before the esports World Championship 2022, Zwift announced that a rider received a 30-day suspension because he changed his weight values during the race (without being detected).^9^ According to Rojas-Valverde, Córdoba-Blanco and González-Salazar (Rojas-Valverde et al., 2022) the lack of clear guidelines in eCycling suggest that these competitions have to go a long way before being considered a real sport.

A crucial difference with other sports is the organization and governance, also in relation to cheating issues. Esports lack a central governance but rather have several competing institutional organizations (Jenny et al., 2017), which implies that there is no uniformity in sanctions. Issues of integrity and ethics are closely related to the model of governance of the eSports industry. According to Peng and colleagues (Peng et al., 2020) the esports network is not governed in a way that it enables it to respond quickly and appropriately to the issues it encounters. Based upon an analysis of the current esports governance model, they plead for more social responsibility of new digital and technological industries and question the “*often-neglected social impact of esports as a result of the profit-seeking governance model*.” Given the integrity issues that esports are facing, governments should play a main role in facilitating another model of governance. “*Given that organizations rarely involve responsibility or ethics officers, further work should also clarify game development processes and propose how to embed ethical analysis into the development work itself*.” [(Hyrynsalmi et al., 2021), p. 7].

Several governance reforms are proposed in recent publications, to address the potential harms and the wellbeing of vulnerable, young participants (Kelly et al., 2022). More focus and engagement should be directed toward the responsibilities of different stakeholders in helping ensure eSports players' mental and physical health and to ensure a safe environment for players that pursue a professional career (Hong, 2022). At the same time, research is also needed to gain insights into the potential positive role and impact of esports in society, including beneficial socializing, pedagogical or educational aspects (Riatti and Thiel, 2021).

## The Value of a Level Playing Field

Having a level playing field is about having a fair and open competition–meaning that all athletes should enjoy the same opportunities in terms of sports equipment, training facilities and coaching. Unfortunately, it seems, this is a utopian dream for traditional sports and esports alike. In this section, we will reflect on “cost”, “access” and “unfair advantage” (Dyer, 2020) to investigate how (upcoming) esports compare to traditional sports in offering a level playing field to their athletes.

One of the prevailing objections to commercially popular games joining the ranks of esports, is the practice of needing to buy items with real currency. Players that want to compete at the highest level in “FIFA Ultimate Team” need to spend anywhere between $1,700 and $27,000 on virtual players (often *via* loot boxes) to have a chance at winning (Tregel et al., 2020). In general terms, the need to spend money to gain advantage in serious competition is known as *pay to win* (cf. (Tregel et al., 2020; Zendle et al., 2020). When tied to lootboxes–virtual items with unknown or later revealed value–this is frowned upon by the competitive gaming industry (Tregel et al., 2020). Indeed, loot boxes can be seen as a form of gambling (Drummond et al., 2020)–causing some countries to ban or regulate loot-box practices. Pay to win can also be seen in the rate at which players need to update their game. Games like FIFA have yearly update-cycles which require users to (a) buy a new instance of the game and (b) reassemble a new team to compete with the latest version. Clearly, quite some money can be spent on playing games.

Although it seems odd to think of traditional sports to be influenced by *pay to win*, (top) athletes / sports organizations need to spend quite some money on attire, equipment and other training facilities to compete. Probably the most clear cut examples stem from F1-racing and soccer. In F1-racing, hefty sums of money are spent on R&D, racing equipment and salaries. And to get the best players in soccer, incredible amounts of transfer money are paid, even up to €222 million for Neymar in 2017. Also on the (amateur) individual level, pay to win is apparent. With the introduction of the Nike Alpha Fly shoes, Nike created a shoe that was able to reduce the overall metabolic cost in running by 4% (Hoogkamer et al., 2018). This might not seem like much, but over the course of a marathon it can make a substantial difference. The catch? Nike's revolutionary new shoes cost about 400–500% more than conventional running shoes (Dyer, 2020). Similar arguments can be made for cycling, rowing and other sports. In extrema, consider the use (and cost) of low-oxygen chambers and hyperbaric oxygen therapy. Pay to win is even visible at a global scale–the gross domestic product of a country is directly reflected in the medal table of the Olympics (Bernard and Busse, 2004; Schlembach et al., 2022). Indeed, “money can buy gold” (James, 2010).

Another consideration in the level-playing-field discussion is accessibility–are technologies, resources and facilities equitable in terms of availability? While related to cost, the issue of availability is also clearly distinct. Sponsorships, contracts, patents and logistics might limit athletes' access to relevant resources. Esports appear to be quite equitable in this regard. Games are designed and distributed to be played by as many people as possible and through the internet most resources can be purchased with the click of a button. Every player that would want to buy a certain (virtual) item, would be able to do so (however, refer to the loot-box issue). There are however some interesting exceptions from this general trend. Recently, high-end graphics cards are hard to come by because of a global shortage in silicon chips. With faster graphics cards delivering high-detail images with stable and higher refresh rates, players with access to such cards have a performative advantage over players who do not. Other exceptions are seen in the form of exclusive training facilities, such as Fnatic's *High Performance Unit*- a training institute for Fnatic-athletes.

In traditional sports access to technology might be limited due to sponsorships, contracts and patents. One recent example of that can be found with Nike, who sponsored Eliud Kipchoge with prototypical running shoes. The shoes, which were later commercialized under the names Nike AlphaFly and Nike VaporFly, were at the time limitedly available. In light of the previously mentioned reductions in metabolic cost, this is controversial (Sailors, 2019; Rosenberg and Sailors, 2021). Another example stems from the development of the clap-skate, a Dutch invention for speed skating (van Ingen Schenau et al., 1996). While met with skepticism upon introduction, the clap skate quickly gained popularity; first among the Dutch female top skaters and later among the men. Once its potential for the sport became apparen, the Dutch frustrated the wider distribution of the clap skate, limiting its availability to the competition during the 1998 Winter Olympics of Nagano (Van Hilvoorde et al., 2007). Other such examples can be derived from swimming, Formula 1 racing and cycling.

As outlined above, excess costs and limited access or availability might cause an unfair advantage for athletes in both traditional sports and esports. To level the playing field, costs for competing should be fair and the availability of technologies equitable. We argue that esports holds just as much potential (if not more) to allow for a level playing field as traditional sports.

## The Value of Inclusivity

The International Olympic Committee holds the value of inclusion in high regard. From the Olympic Charter it can be read that: “*The practice of sport is a human right. Every individual must have the possibility of practicing sport, without discrimination of any kind and in the Olympic spirit, which requires mutual understanding with a spirit of friendship, solidarity and fair play.”* [Rosenberg and Sailors, 2021), p. 8]. Indeed, in many ways, the Olympic Movement seeks to offer a podium to as many athletes as possible, making competitive distinctions based on sex, physical ability (Paralympics), and mental ability (Special Olympics). The level of distinction can be quite fine-grained. To do right to all Paralympic athletes, the 2020 Paralympics featured a staggering 539 events in 22 sports. Arguably though, having that many sub-competitions is not the same as having an inclusive sports climate. Nor is it the same as having a level playing field. Track athletes with a visual impairment (T11-13) do not directly compete with track athletes with cerebral palsy (T31-38). Indeed, many athletes that practice the same sport will never cross swords at the Paralympics. This is where esports may have an exemplary role to fulfill, outperforming its traditional counterpart.

Through game balancing [e.g., (Mueller et al., 2012; Gerling et al., 2014; Altimira et al., 2016; Jensen and Grønbæk, 2016) and the design of ranging input devices [e.g., (Gerling et al., 2016; Graf et al., 2019], a much wider range of people can be brought together to play the *same* field. Due to the digital-physical nature of esports and games, input and output can be creatively but fairly remapped to accommodate players with ranging physical (and even mental) abilities. The graphic design of games can be adapted to accommodate people with visual impairments. And for many esports, sex might not even be an issue from a physical point of view. As such, esports and game design have an important role to play to create an open and inclusive arena–creating an *a priori* level playing field, rather than an *a posteriori* one.

## Ameliorating Value-Tensions in Esports

Our investigation of the values of *physicality, meritocracy, competition, fair play, level playing field* and *inclusion* revealed that esports champion much of the same values that traditional sports do. Indeed, many aspects appear to be shared between the two. Yet, where traditional sports are celebrated for many of their attribute values, esports are not typically lauded for their psychosocial benefits or for their pristine competitive nature. Instead, the image of esports is muddled by negative values such as violence and game-addiction. And while such values are not foreign to traditional sports either (e.g., Freimuth et al., 2011), they do tend to overshadow the benefits of esports and gaming (Granic et al., 2014). To point out future directions to ameliorate and resolve some of the value-tensions that keep esports from ascending the Olympic podium, we borrow from *Value Sensitive Design* (Friedman and Hendry, 2019). Value Sensitive Design is “*a theoretically grounded approach to the design of technology that accounts for human values in a principled and comprehensive manner throughout the design process*.” [(Friedman et al., 2002), p. 1].

In addressing value tensions Friedman and Hendry (2019) mention two overarching strategies: a focus on ‘shared action, not reasons’ and when in ‘significant doubt: *wait*’. It seems we are currently in the second state where esports are being considered but not actively denied or accepted. Here, the decision to omit action is important. It offers room for the emergence of new ideas and technologies; furthermore, it allows the socio-politic context to shift (Friedman and Hendry, 2019). Regarding the shared actions strategies, the design and features can be agreed upon although there are very different reasons to do so. For instance, we imagine in our context game companies might be interested in certain esports being part of the Olympics for visibility in order to make *profit*. Whereas IOC might be interested because of the *inclusion* of new generations of sporters and viewers. For the athletes themselves, being able to play at the Olympics -like any Olympic sporter- offers the *prowess* facilitated by participation in the event and public recognition. An action can thus be the same even when the underlying reasons can be very different.

In the description of the VSD methodology framework so far, we have mostly taken a *retrospective* view looking at current possibilities and practices but do now also turn toward a *proactive design* perspective to suggest how technology (including policies) might be changed or selected to overcome value tensions. As Friedman and Hendry mention: “*proactive work benefits from examining how related technologies have progressed, what proximate and distal causes have led to sociotechnical failures, and what assumptions are not likely to hold in the future with particular attention to inflection points.”* [(Bernard and Busse, 2004), p. 89].

We do this in two ways, we first briefly relate our analysis to the explicitly mentioned values and practices of the IOC, and then discuss tensions which also relate to recorded statements of its current president and the IOC Olympic Agenda 2020+5 as well as several scholars looking into the similar question. Building on the history of the Olympics and Olympic Movement it is good to first relate our previous analysis to their three key values, the first is related to our discussion of meritocracy under the term *Excellence*, “*reaching one's personal objectives with determination in the effort…and benefiting from the combination of a strong body, will and mind”* ^10^. Ideally this highlights participation and the athlete's own objectives and maximum effort rather than just winning, however in our previous analysis we do also see competition as an important value in current practices. The second value is closely related to our discussion of fair play extended with sustainability: *Respect, “respect for one another, for rules and for the environment”*. The third value relates to our discussion of inclusion but also building toward a peaceful world: *Friendship*.

Based on the additional values we indicated about a level playing field and fair play's relation to governance, this leads us to highlight a brief proactive discussion around three value tensions seemingly obstructing further inclusion of esports: peace and conflict, physicality and virtual empowerment, and governance and profit.

### Peace and Conflict

On the one hand, peace is highlighted under IOC's value of Friendship and also actively targeted with the tradition and symbolism related to the Olympic Truce. On the other hand, the most popular esports seem to realize their value of conflict with first-person shooters and war themed strategy games (Jenny et al., 2017). This tension is according to Bach also a main reason to exclude these popular esports: ‘*e-games which are killer games or where you have promotion of violence or any kind of discrimination as a content... they can never be recognized as part of the Olympic movement.’*^11^ The current approach is to simply exclude certain esports on the basis of their theme when they contrast the core values and to consider inclusion otherwise. Instead, as Heere (Heere, 2018) suggests certain sports games might serve as a gateway to a more active life promoting Olympic values through their themes. This direction of promoting Olympic values through games is also included in the Olympic Agenda 2020+5, where the IOC recommends to: “*Encourage the development of virtual sports and further engage with video gaming communities. Leverage the growing popularity of virtual sport to promote the Olympic Movement, Olympic values, sports participation*”.

In itself the complaint of a non-peaceful theme might be at odds with the inclusion of traditional sports such as shooting in almost all modern games. Taking VSD's suggested examination of related progressions, we can try to build on the progression that the shooting events made from shooting live animals toward shooting targets and skeets. Perhaps if the players themselves would be visualized as walking targets or if the aim is to shoot at non-living targets rather than human virtual characters this could give a less violence or war-oriented association. Or alternatively, if the act of shooting is countering the earlier mentioned IOC value related to peace, the same game mechanics might be addressed with other visualizations or themes, so for instance a competitive game can be designed to aim wands and trigger magic spells at another virtually moving player.

Still, peace is about more than not shooting and a game's theme can conflict in other ways with Olympic values. Many shooter games draw inspiration from geopolitical conflicts. For such shooter games to be considered by the IOC in the Olympic program, non-neutral political stances about terrorism, East versus West, and other historically real-world relations embedded in the games need to be addressed. Whether such a redesign / recontextualization would be accepted by a broader (e)-athlete and spectator audience remains to be seen (Miah, 2021).

### Physicality and Virtual Empowerment

Regarding the implementation of game mechanics, we previously argued that many forms of esports exist and that there is no reason they need to be any less physical than their traditional counterparts. Building on the granularity of physical action, many scholars, including Jenny et al. (2017) and Parry (2019), do argue the current cut-off is set to exclude esports. We only agree that esports thrive by the fact that even small amounts of physical actions provide lots of feedback. To balance fun and realism there is a form of “virtual empowerment”, an exaggerated feedback of actions where for instance a normal jump is translated to a visualized five meter high jump on a screen (Hämäläinen et al., 2005) or button presses and mouse clicks mapped to running around in a virtual environment.

In the most extreme version any form of input translated into a virtual domain could be argued to exclude it as a potential Olympic sport, or to come to a similar conclusion by making an analogy of this virtual empowerment with the explicit exclusion of motorized sports (Parry, 2019). If the lack of gross motor movements (Jenny et al., 2017) or “whole-body skills” (Parry, 2019) are hindering esports from joining the ranks of sports, then physical virtual sports with ergometers such as Zwift's cycling or rowing would be a direction to overcome this tension. Indeed even Parry (2021) agrees that this form of turning physical action into the virtual domain might well be “real sports”. If however, as we discussed according to Gentile's taxonomy (Gentile, 1972, 1987), the lack of moving through the physical space is a reason to exclude esports, then the possible rise of Virtual Reality Sports^12^ and other earlier mentioned location-based esports still provide interesting alternatives. In an earlier mentioned news item, quoting Bach, they also stated:“*They show physical activity which can be compared to physical activity in some traditional sports, in order to be recognized by the IOC and by the Olympic movement, it is not enough just to show physical activity there. The physical activity must also be in compliance with the values of the Olympic movement”*. Note that the overlap in skill requirements with sports such as shooting and archery are at the same time used as a counter argument by scholars, for instance that only two sports are not using gross motor skill (Jenny et al., 2017). Therefore, from a proactive perspective it could provide clarity if IOC would further explicate or appropriate and communicate a task complexity framework beyond the exclusion of ‘motorized' and ‘mind sports' as a basis from which to exclude certain sports (both esports and traditional sports) in relation to a seemingly persisting physicality argument.

### Fair Play Through Governance and Profit Through Design

Besides the nature of the activity and the physical characteristics, we reiterate the importance of governance on the International Federation level as an important criterion. Whereas esports are primarily owned by companies aiming for profit, sports are not owned but governed (Parry, 2019). In our analysis we indicated the limited or total lack of governance and accompanying institutionalization as an issue. The current state of affairs in terms of esports governance is taken either as an argument to exclude it from sports (Parry, 2019) or to embrace esports as a manifestation of sportification (Heere, 2018). Moreover, without a governing body there will be no Olympic inclusion–the IOC requires proposed events to be related to a recognized international federation.

If we accept that the current self-organized game-developer based governance is insufficient for inclusion at the Olympic level, we foresee three main lines in which this tension could be addressed. The first would be to have a self-organized esports organization representing esports with a variety of games (e.g., the International Esports Federation, the World Esports Association, and/or the Global Esports Federation). The second would be a grassroot, broadcaster, or event-based organization where sets of games might have their own representing body. The third would be to govern by adoption. Traditional sports organizations could adopt esports disciplines that embody much of the same values, themes, and behaviors as their original target sport.

According to Miah [(James, 2010), p. 15], Thomas Bach indicated the latter as a possible way to resolve this tension: ‘*we are encouraging their International Federations to look into the e-versions of their sport and to try and get, at least, a regulatory authority, so that then we would have a partner there with whom we could drive this development'*. For all these forms the power-relation between the organization (e.g. International Federation) and designers of the e-version of a sport is important as Bach mentioned. The consideration whether to include a game could also be dependent on this as it might relate to the guarantees toward long term viability of the sport. In this line of power, it is also important to look at the relation between the commercial game and the esports activities. Some games invest more toward their esports communities than others. For instance, Pro Evolution Soccer recently rebranded to eFootball and highlighted their interest in esports^13^ Another issue related to the power relations is the governing of governing bodies, in line with Bach's statement UCI also refuses to recognize any world governing esports body (Parry, 2021).

Finally, even if there is an institutionalized form and enough power to govern the esport then the question remains whether the athletes themselves actually see their values represented and still want to be associated with a sportified version under that “new” governing body. Value tensions might arise as a direct result of the process of “sportification”. As a case in point, consider skateboarding (Batuev and Robinson, 2017, 2018), part of the Olympic program in Tokyo 2020. The anti-establishment attitude that runs through skateboarding culture runs counter to the commercialization and institutionalization that is associated with the Olympics. “*The organizational arrangements currently look like a compromise between the values of the Olympic movement and the skateboarding community, such as the full institutionalization of sport vs. retaining a fair degree of anti-establishment flexibility; formality of sport regulations vs. creativity of expression in skateboarding; and strict judging criteria vs. choosing the personal favorite*.” [(Friedman and Hendry, 2019), p. 20]. In a similar fashion some surfing and parkour athletes contested the competitive nature that was required to “sportify” their activity with accompanying institutionalization toward Olympic inclusion (Parry, 2019).

## Discussion

We have tried to align and contrast existing values in sports with those current in esports. However, this practice goes beyond the fact that values are dynamic–subject to change. Building on the Value Sensitive Design framework we argue a continuing discussion is to be held about developments related to the values to be promoted. In this discussion it is important to work closer together with a variety of stakeholders including not only IOC-perspectives but also esport athletes, broadcasters and commentators, coaches, existing candidate institutions, spectators, and game development companies. For instance, to answer the question “What value-oriented criteria will be used to judge success of design?” and “How do policies, laws, or regulations create opportunities or constrain options for technological development?” [(Bernard and Busse, 2004), p. 32, 34]. Which way do we want them to go and why? And how might esports contribute to (or take away from) that? At the end of the day, esports are malleable–through design, esports can be what we want it to be.

Regardless of the formal status of esports, new digital expressions of gaming behavior have become an integral part of Asian and Western sports culture. As a result of the Covid-19 pandemic, this process of digitization has accelerated, spurring new systems that blur the lines between esports and traditional sports (Postma et al., 2022). Various scholars investigated how the Covid-19 pandemic makes us rethink the relation between legacy sports and esports and accompanying tensions and opportunities regarding governance, participation, and spectatorship (Manoli et al., 2022). Twitch for example, a popular live streaming platform with a focus on video games, saw a significant rise in viewership during the pandemic (Leith and Gheen, 2021). This, and other cultural currents, have now led to a discussion within the IOC as to whether esports can actually be admitted to the Olympic family of sports (Miah, 2021). In this discussion, 'exemplary' variants are mentioned, such as cycling on the Zwift platform, which should illustrate the Olympic credibility and potential of esport.

This raises various questions about the way in which this choice is legitimized. It seems that the IOC is targeting precisely those digital variants that have the most similarities with the sport as we know it in terms of motor skills and challenges. In the case of Zwift, that is obvious, namely cycling. This choice could also open the door to comparable digital reflections of traditional (mostly cyclical, individual) sports, such as virtual rowing or running. There is something to be said for this choice, but we also present serious objections. Based on an analysis of the fundamental characteristics and values of sport, we argue that there is also a risk of unwarranted selectivity here, but also of a missed opportunity, given the potential richness of (new) esports.

In our argument, we make critical comments about a demarcation between 'good' and 'bad' esports, based on criteria such as physicality or similarities with existing sports. In our plea, we make it clear that it is more about the intrinsic characteristics of the playful activities that are important, such as the meritocratic ideal, competition, level playing field and fair play. These characteristics are closely related to the governance of sport, and the way in which these characteristics are safeguarded and the way in which cheating and the creation of equal opportunities are dealt with.

For the IOC, it is important that intrinsic sport values such as the meritocratic ideal and the level playing field are carefully safeguarded, and that the organizational structure of the competition guarantees a competition that is in line with Olympic values. The current partnership between the UCI and Zwift demonstrates this commitment to good governance, but at the same time illustrates the crucial steps that still need to be taken to ensure a fair competition that is attractive and credible to a wider audience.

For esports themselves, the question is also on the table to what extent the Olympic status is desired, and what that status means for their own identity and culture. The governance of Olympic sports also goes hand in hand with forms of surveillance (think of doping control). This inclusion will irrevocably lead to structural and organizational adjustments aimed at increasing the chances of winning Olympic medals (think of talent identification). Although there are already talent schools for esporters in many parts of the world, this phenomenon will also meet social resistance, especially when commercial interests prevail over the pedagogical and moral issues it raises.

When it comes to joining the family of Olympic sports, it is also about an accurate analysis of the Olympic values and the way in which they are propagated or ignored in a virtual world. An example of this is inclusivity. Esports have a lot of potential when it comes to reaching and including diverse groups of people [e.g. (Graf et al., 2019)], who within traditional sports are more likely to encounter exclusion due to, for example, physical limitations or stigmas. Esports are malleable and technology ever-advancing. As such, the inclusion of esports in the Olympic program appears to be just a matter of time. The question is, when the time comes, what values would we like to see reflected in a debuting Olympic esport? Ultimately, it is up to the IOC to decide, so we ask once more: Dear IOC, what would you like your first esport to look like?

## Author Contributions

All authors contributed equally to the conception, creation of the manuscript, manuscript revision, read, and approved the submitted version.

## Funding

This research was partially made possible in the context of the national funded ZonMW Smart Sports Exercises Project - grant number 546001004.

## Conflict of Interest

The authors declare that the research was conducted in the absence of any commercial or financial relationships that could be construed as a potential conflict of interest.

## Publisher's Note

All claims expressed in this article are solely those of the authors and do not necessarily represent those of their affiliated organizations, or those of the publisher, the editors and the reviewers. Any product that may be evaluated in this article, or claim that may be made by its manufacturer, is not guaranteed or endorsed by the publisher.

## Footnotes

^1^International Olympic Committee (2021) IOC executive board proposes Olympic Agenda 2020+5 as the strategic roadmap to 2025.

^2^Interestingly, task complexity, according to Gentile, would decrease when users move from mouse and keyboard to computer-vision input, such as with the Kinect for XBox and the EyeToy for PlayStation.

^3^ https://www.arcadia.tv

^4^Interestingly, there are no rules prohibiting a rider to draft for extended periods of time without rotating position. However, it *is* considered bad etiquette / sportsmanship to do so.

^5^ https://www.wired.com/story/best-minecraft-builds/

^6^Competitive balance is core to the economics of professional sports (Rottenberg, 1956). Balanced competitions generate more excitement, fan interest, and revenue than unbalanced competitions (Neale, 1964). Interestingly, much of the economic principles that apply to professional team sports also apply to esports (Scelles et al., 2021).

^7^*British Cycling Strips Zwift Championship Winner for Cheating | DC Rainmaker*.

^8^*Disqualified British Champion and Banned from Racing - My response - YouTube*.

^9^*Zwift Bans Cheat Whistleblower: A Deeper Dive Into the Issue | DC Rainmaker*.

^10^https://olympics.com/ioc/faq/olympism-and-the-olympic-movement/what-are-the-olympic-values and by The Olympic Museum Educational and Cultural Services in 2013 explaining these values, the motto, and overall vision of the Olympic movement https://olympics.com/museum/visit/schools/teaching-resources/support-de-cours/olympism-and-the-olympic-movement last accessed 11-3-2022.

^11^According to this news item quoting Bach https://www.reuters.com/article/olympics-ioc-bach-idUSL3N10K089, last accessed 14-3-2022.

^12^Consider for instance, Arcadia (https://www.arcadia.tv/), a digital-physical VR-setup where athletes move through physical space in order to move through digital space. Athletes play with head mounted displays and the audience can also see the in-game content. Or consider BeatSaber World Cup events, where participants compete in VR on a game that requires gross motor movement (https://cube.community/main/tournament/bswc2021/info#links).

^13^In their Steam release notes they include direct mention of eSports including a value related move toward equal cross-platform access https://store.steampowered.com/app/1665460/eFootball_2022.
